# 1例ALK阳性肺肉瘤样癌报道

**DOI:** 10.3779/j.issn.1009-3419.2017.05.11

**Published:** 2017-05-20

**Authors:** 晔红 缪, 琳 凌, 秀琴 张, 建安 黄

**Affiliations:** 215006 苏州，苏州大学附属第一医院呼吸内科 Department of Respiratory Medicine, the First Affiliated Hospital of Suzhou University, Suzhou 215006, China

肺肉瘤样癌（pulmonary sarcomatoid carcinoma, PSC）是一类罕见的含有肉瘤形态细胞或肉瘤样分化的非小细胞肺癌（non-small cell lung cancer, NSCLC），在所有肺癌中所占比例为0.1%-0.4%^[1]^。PSC恶性程度高，预后差，平均生存期仅13.3个月，低于其他类型NSCLC^[2]^。PSC对放化疗不敏感，首选治疗方法为手术治疗，但多数患者发现时已处于疾病晚期，失去手术指征，且术后复发率较高，故治疗存在一定难度。有关靶向药物对PSC的治疗效果目前尚不明朗且相关报道很少。本院近期收治了1例间变性淋巴瘤激酶（anaplastic lymphoma kinase, ALK）阳性PSC患者，克唑替尼联合放化疗治疗3个月后疾病获得明显缓解，现报道如下。

## 1 病例报告

一般资料：患者男性，31岁，因“胸闷伴咳嗽1周”入院。患者1周前无明显诱因下出现活动后胸闷胸痛，伴少许咳嗽，无咳痰，同时出现右眼视物模糊伴右面部感觉减退，遂至我院门诊就诊，行胸部计算机断层扫描（computed tomography, CT）示：左侧肺门占位；右肺下叶纤维灶；双侧少量胸腔积液（图 1）。患者既往体健，否认高血压、糖尿病等慢性病史，否认粉尘及放射性毒物接触史，否认烟酒嗜好，否认传染病及家族型遗传病病史。

**1:**
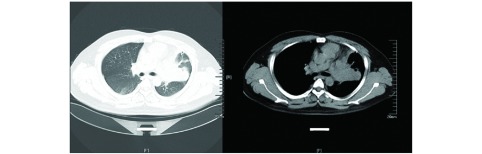
胸部CT（2016-11-12）：左肺门软组织影，大小约7.8 cm×8.8 cm，分叶状，密度尚均匀，周围可见肿大淋巴结影。 Chest CT scan (2016-11-12): A lobulated soft tissue giant tumor located in the left pulmonary hilum with the size of about 7.8 cm×8.8 cm showed homogeneous density; lymphadenopathy around it can be seen. CT: computed tomography.

入院查体：神志清楚，精神尚可，右眼上睑下垂，眼裂高度0 mm，右眼视力0.15，左眼视力0.6，瞳孔圆，右眼对光反射（-），左眼对光反射（+）。左肺呼吸音低。余查体无特殊。

入院后辅助检查：血尿粪常规、生化全套无明显异常；肿瘤全套：CYFRA211 3.52 ng/mL，糖类抗原CA199 408.04 U/mL，癌胚抗原CEA 1.73 ng/mL，糖类抗原CA125 183.1 U/mL，SCCA 2.7 ng/mL，NSE 21.47 ng/mL，CA72-4 1.02 U/mL；心超、心电图无明显异常；腹部B超：肝右叶低回声，大小约24 mm×18 mm，考虑转移；头颅核磁共振成像（magnetic resonance imaging, MRI）增强扫描：中颅窝底骨质异常，考虑转移；骨扫描：右侧锁骨中段、右侧骶髂关节反应性骨形成活跃，考虑转移。

结合病史及相关检查，考虑患者为晚期肺癌，行纤维支气管镜检查，镜下可见：左上叶开口见新生物阻塞管腔，表面有出血坏死组织附着（图 2）。纤维支气管镜活检免疫病理诊断为肺肉瘤样癌，肿瘤细胞Vmentin（+），CK（+），Ki-67（+，50%），CK7（-），TTF-1（-），NapsinA（-），CK5/6（-），P63（-），P40（-），CD56（-），CgA（-），NSE（-），HMB45（-），S-100（-），CK20（-），LCA（-），Desmin（-），ALK（D5F3）（强+），对照组阴性（图 3）。

**2:**
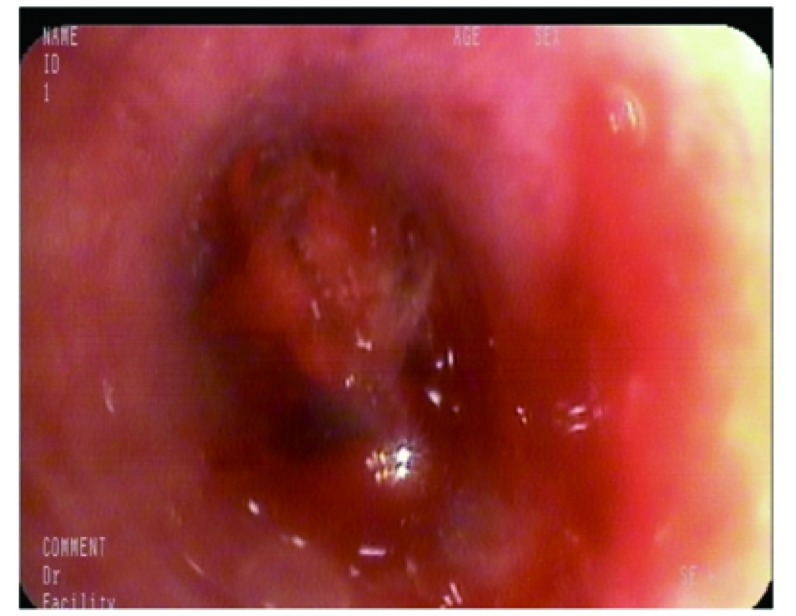
气管镜：左上叶开口处见新生物阻塞管腔，表面有出血坏死组织附着。 Bronchoscopy showed that neoplasm blocked the left upper lobe bronchus openings, hemorrhage and necrosis can be seen on the surface.

**3:**
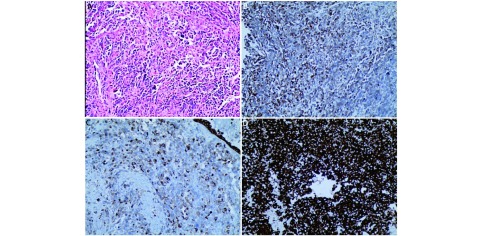
病理。A：肿瘤细胞中等大小，细胞异型性明显（HE×200）；B、C、D：免疫组化[B：vmentin(+)；C：ck(+)；D：ALK(D5F3)(+)]（免疫组化采用罗氏全自动染色仪染色×200）。 Pathology. A: Tumor cells are median size with marked atypia (HE×200); B, C, D: Immunocytochemistry [B:vmentin(+), C:ck(+), D:ALK(D5F3)(+)](Roche automatic electrochemiluminescence immuno-assay analyzer ×200).

治疗：患者一般情况可，体力状况（performance status, PS）评分1分，诊断肺肉瘤样癌Ⅳ期，为最大程度改善预后，经病人知情同意后，2016-11-25、2016-12-21、2017-01-17、2017-02-18行多西他赛130 mg d1+奈达铂130 mg d1化疗4次，每次化疗结束后续以克唑替尼250 mg bid口服治疗，同时行根治性放疗，具体为X-Ray 6 MV IMRT DT:计划肿瘤靶区（planning gross tumor volume, PGTV）（海绵窦、蝶窦大体肿瘤）：66 Gy/32 f，计划靶区（planning target volume, PTV）（包括PGTV及周围亚临床灶）：60 Gy/32 f，同时予“希美纳1.25 g d1、d3、d5 qw”口服以增敏，截至目前已放疗25次。

随访：患者定期入院，每次入院均复查3大常规、生化全套、肿瘤全套、心电图等，隔次入院复查胸部CT及头颅MRI，患者胸闷、胸痛、咳嗽等症状均较前明显好转，无明显恶心、呕吐、腹泻、便秘、视觉改变、乏力、水肿等克唑替尼不良反应，最近一次入院时间为2017-02-17，查体：Horner征改善，右眼上睑下垂较前好转，眼裂高度5.40 mm，右眼视力0.4，左眼视力0.6，瞳孔圆，右眼对光反射（+），左眼对光反射（+）。左肺呼吸音减弱较前明显好转。辅助检查：血尿粪常规、生化全套、心电图无明显异常；肿瘤全套：CYFRA211 3.52 ng/mL，CEA 4.12 ng/mL，CA125 32 U/mL，NSE 11.58 ng/mL，CA72-4 4.46 U/mL；腹部B超提示肝内病灶较前缩小，大小约18 mm×11 mm。患者近两次胸部CT（图 4，图 5）提示病灶每次均较前明显吸收，但头颅转移灶无明显改变。

**4:**
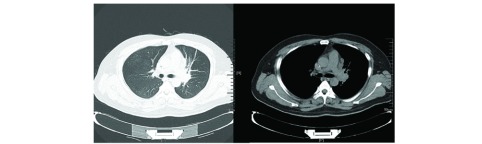
胸部CT（2016-12-14）：左肺门软组织影，大小约4.4 cm×2.6 cm，分叶状，密度尚均匀，周围可见肿大淋巴结影。 Chest CT scan (2016-11-12): A lobulated soft tissue giant tumor located in the left pulmonary hilum with the size of about 4.4 cm×2.6 cm showed homogeneous density; lymphadenopathy around it can be seen.

**5:**
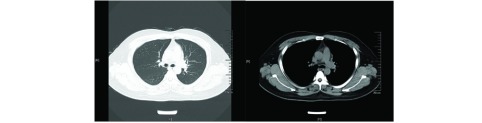
胸部CT（2017-02-17）：左肺门软组织影，大小约3.2 cm×2.4 cm，分叶状，密度尚均匀，周围可见肿大淋巴结影。 Chest CT scan (2017-02-17): A lobulated soft tissue giant tumor located in the left pulmonary hilum with the size of about 3.2 cm×2.4 cm showed homogeneous density; lymphadenopathy around it can be seen.

## 2 讨论

PSC在2004年版世界卫生组织（World Health Organization, WHO）肺肿瘤组织学分类中首次被定义为一类分化差的NSCLC，包括5种亚型：多形性癌、梭形细胞癌、巨细胞癌、癌肉瘤、肺母细胞瘤。PSC多见于中老年吸烟男性，临床表现及影像学表现无特异性，与肿瘤发生部位相关，多数患者因咳嗽、胸痛、咯血等呼吸道症状就诊，少数患者以声音嘶哑、肩部疼痛等作为首发症状，本例患者以胸闷咳嗽等为首发表现，并出现Horner综合征等相关体征，符合晚期肺恶性肿瘤的临床表现。

PSC的诊断主要依靠电镜下细胞形态及免疫组织化学染色，既往认为支气管镜活检或痰细胞学检查很难确诊，故PSC的诊断常依靠手术获得病理。但因相当一部分患者发现时已处于肿瘤晚期，手术诊断代价较大，最新指南肯定了小活检的诊断价值^[3]^，本例患者通过支气管镜夹取肿瘤组织，免疫病理最终明确诊断为PSC，避免了手术带来的巨大创伤及经济负担。

PSC侵袭性强，免疫组化常提示上皮性标志物和间叶性标志物混合存在，这可能与PSC上皮间质转化（epithelial-mesenchymal transition, EMT）相关，即PSC的癌成分向肉瘤成分转化，这种表型转换使肿瘤细胞易摆脱细胞间的连接，从而向周围组织、血管、胸壁侵袭^[4]^。本例患者发现时已出现全身多发转移，印证了PSC的强侵袭性。而PSC的强侵袭性必然预示其治疗难度大，研究^[5]^支持晚期PSC患者对一线化疗不敏感，放化疗术后平均生存期仅为2.7个月^[6]^，故积极寻求靶向治疗是改善此类肿瘤预后的较好的方法。

一项纳入33例PSC患者的临床研究^[7]^检测了每例患者的多个基因突变，结果显示24例（72%）患者至少存在一个基因突变，其中19例（58%）检测到TP53突变，10例（30%）检测到*KRAS*突变，而*AKT1*、*JAK3*、*BRAF*、*NRAS*及*PIK3CA*突变各被检测到1例（各3%），此外，ALK重排被检测到1例。另外一项多中心研究^[8]^共纳入141例PSC患者，其中ALK重排患者占3.5%。以上研究肯定了PSC患者中ALK等相关基因突变或重排的存在，而最新指南更是建议根据PSC相关的组织病理类型，对可能存在基因异常的组织进行检测，以指导医生进行个性化的治疗^[3]^，包括小病理组织。这为PSC靶向药物的临床应用提供了依据。目前国内外已有研究肯定了表皮生长因子受体酪氨酸激酶抑制剂（epidermal growth factor receptor tyrosine kinase inhibitors, EGFR-TKIs）在PSC中的治疗价值，而对于克唑替尼疗效的报道很少。

克唑替尼是ALK阳性晚期NSCLC的一线用药，是一种ATP竞争性酪氨酸激酶抑制剂。该药最初是作为肝细胞生长因子受体（c-MET）抑制剂，随后发现除了c-MET之外，其对ALK和ROS这两种蛋白激酶也有抑制作用，而随着新的致癌基因，棘皮动物微管结合样蛋白4（echinoderm microtubule-associated protein-like 4, EML4）-*ALK*融合基因在NSCLC中的发现，克唑替尼在NSCLC中的治疗也正式拉开了序幕。与传统化疗相比，克唑替尼疗效好，中位无进展生存期长，生存率高^[9]^。且最新研究^[10, 11]^表明，存在脑转移的ALK阳性的NSCLC患者使用克唑替尼联合放疗总生存期可高达49.5个月，相比于无脑转移的患者，脑转移的患者更能从克唑替尼治疗中获益。本例患者确诊PSC，免疫组化提示ALK强阳性，考虑基因的互斥性及经费原因，未检测到EGFR、c-MET等突变，每次化疗结束后续予口服克唑替尼治疗，并行头颅立体定向放射治疗，随访至今3个月，每次复查胸部CT及腹部B超肿瘤病灶均逐渐缩小，提示克唑替尼对于治疗PSC有显著效果，但患者头颅转移灶无明显改变，这可能与肿瘤异质性、PSC对于放疗不敏感、克唑替尼作用时间过短等相关。

此外，最新研究表明，除EML4-ALK外，肝细胞生长因子及其受体（HGF /c-MET）信号通路在PSC的发生发展中有很重要的作用，这可能与*c-MET*基因14外显子跳跃缺失相关，*c-MET*基因14外显子跳跃缺失多发生于NSCLC，并以其中的肺肉瘤样癌和腺癌更多见，在肺肉瘤样癌中的发生率可高达22%^[12]^。Ou等^[13]^曾报道1例存在c-MET扩增而非ALK重排的NSCLC患者在使用克唑替尼后获得快速持续缓解的病例，提示克唑替尼作为一种c-MET抑制剂仍需临床的进一步研究。*c-MET*突变有望成为PSC治疗的新靶点，这也进一步肯定了克唑替尼在治疗PSC中的价值。

总之，PSC是一种特殊类型的NSCLC，预后差，目前治疗方法局限，靶向药物亟需更多的临床研究。本例患者ALK强阳性，口服克唑替尼联合放化疗治疗3个月后胸部CT提示原发病灶明显缩小，这让我们相信对于晚期PSC患者克唑替尼不失为一种好的选择，当然，本例临床研究因病例数较少，未检测*c-MET*及*ROS*突变，联合使用放化疗，研究时间较短，故存在一定缺陷，是否克唑替尼真的能为晚期PSC的治疗提供新的方向，这需要更多大规模的临床试验证实。
